# Eculizumab and ravulizumab clinical trial and real-world pharmacovigilance of meningococcal infections across indications

**DOI:** 10.1371/journal.pone.0332073

**Published:** 2025-09-12

**Authors:** Cynthia Carrillo Infante, Arshad Mujeebuddin

**Affiliations:** Global Patient Safety, Alexion, AstraZeneca Rare Disease, Boston, Massachusetts, United States of America; Keio University School of Medicine, JAPAN

## Abstract

**Introduction:**

Complement component 5 inhibitor therapies (C5ITs) for rare hematological, renal, and neurological diseases are associated with increased meningococcal infection risk. Robust risk mitigation measures include vaccination, drug safety programs, patient safety cards, and antibiotic prophylaxis initiation when starting C5ITs. Here we describe the exposure-adjusted meningococcal infection and mortality rates in eculizumab- or ravulizumab-treated patients based on clinical trial and real-world pharmacovigilance data.

**Methods:**

Global clinical trial and real-world safety data regarding eculizumab and ravulizumab use across indications were recorded in the Alexion pharmacovigilance database. Data for eculizumab (March 2007-October 2024) and ravulizumab (December 2018-December 2024) were searched based on the *Medical Dictionary for Regulatory Activities* (versions 26.1, 27.0, and 27.1) High-Level Term of *Neisseria* infection. Only cases associated with *Neisseria meningitidis* were included. Reporting rates were calculated cumulatively per 100 patient-years (PY).

**Results:**

At the time of analysis, cumulative exposure across clinical trial and real-world settings were 2457 and 91,052 PY, respectively, for eculizumab and 3287 and 34,582 PY, respectively, for ravulizumab. The cumulative meningococcal infection rate in clinical trials was 0.28 and 0.18 per 100 PY for eculizumab and ravulizumab, respectively. Real-world cumulative meningococcal infection rates in patients treated with eculizumab have decreased since 2007 (0.25 per 100 PY in 2024). In patients treated with ravulizumab, the real-world cumulative rate of meningococcal infection remains low (0.10 per 100 PY in 2024). The rates of meningococcal-associated mortality were ≤0.03 per 100 PY in both eculizumab- and ravulizumab-treated patients in clinical trials and real-world settings.

**Conclusions:**

Meningococcal infection and mortality reporting rates have remained stable despite increasing cumulative eculizumab and ravulizumab exposure over time across indications, including rare neurological indications. Infection awareness, existing risk mitigation strategies, and availability of vaccines have effectively reduced the risk of meningococcal infections in C5IT-treated patients, underlining the importance of adhering to those measures.

## Introduction

Rare chronic diseases, such as paroxysmal nocturnal hemoglobinuria (PNH), atypical hemolytic uremic syndrome (aHUS), generalized myasthenia gravis (gMG), and anti-aquaporin-4 antibody-positive (AQP4-Ab+) neuromyelitis optica spectrum disorder (NMOSD) are caused by uncontrolled complement activation [1–4]. Without proper treatment, patients are at risk for significant complications such as intravascular hemolysis and thrombosis in PNH, end-stage renal disease in aHUS, muscle weakness in gMG, and irreversible neurological disability in NMOSD [1–4]. One treatment strategy has been the inhibition of the terminal effector of the complement pathway, complement component 5 (C5), which prevents the formation of the terminal complement complex while preserving critical functions of upstream components that mediate pathogen opsonization and immune complex clearance [5–7]. Eculizumab and ravulizumab are humanized monoclonal antibodies that bind to C5 [5,6] with established safety and efficacy in patients with PNH, aHUS, gMG, and AQP4-Ab+ NMOSD in several clinical trials [8–12]. From their initial approvals in March 2007 and December 2018, respectively, eculizumab and ravulizumab have been approved in 46–64 and 61–70 countries for the treatment of these diseases.

The complement system is critical for innate immunity and responsible for eliminating pathogens such as *Streptococcus pneumoniae*, *Haemophilus influenzae*, and *Neisseria* species including *Neisseria meningitidis* (*Nm*) through cell lysis and/or phagocytosis [13,14]. Patients with proximal complement deficiencies have an increased risk of infection by nonmeningococcal encapsulated bacteria, whereas terminal complement deficiencies and complement inhibitor therapies are associated with an increased risk of being infected by *Nm* [13–15]. Historically, patients with a terminal complement deficiency who had invasive meningococcal disease were less likely to succumb to the infection compared with the general population [16]. Given the known increased risk of infection, an enhanced postmarketing pharmacovigilance surveillance was established for eculizumab and ravulizumab to collect data across a larger patient population to ascertain the safety profile of both treatments. To minimize the risk of meningococcal disease when starting complement-inhibiting therapies, several mitigation strategies are in place, including meningococcal vaccination, drug safety programs, safety cards and educational materials for patients and physicians, and the use of antibiotic prophylaxis in patients who are not yet fully vaccinated until 2 weeks after vaccination or per local guidelines. As availability of vaccines against specific meningococcal serogroups (e.g., A, C, W, Y, MenB), guidance for boosters, and protocols for antibiotic prophylaxis vary by country/region, local guidelines and local/regional product information for eculizumab and ravulizumab should be consulted for specific guidance on the mitigation of meningococcal infection.

The objective of this analysis is to summarize the exposure-adjusted meningococcal infection and mortality rates in patients treated with eculizumab and ravulizumab based on clinical trial and real-world pharmacovigilance data.

## Methods

Global clinical trial and real-world safety data regarding eculizumab and ravulizumab use in the treatment of PNH, aHUS, gMG, and NMOSD were collected and recorded in the Alexion pharmacovigilance database. Eculizumab data were analyzed from March 16, 2007, (the date of first marketing authorization) through October 1, 2024. Ravulizumab data were analyzed from December 21, 2018, (the date of first marketing authorization) through December 31, 2024. Both solicited and spontaneous adverse events were included and may have been identified through review of individual case safety reports (ICSRs), published articles and conference abstracts, results from Alexion-sponsored trials and externally sponsored scientific research, reports from the product complaints management systems, regular analysis of aggregated ICSR data, or safety-related inquiries from health authorities, healthcare providers, and consumers. The pharmacological database was searched based on the *Medical Dictionary for Regulatory Activities* versions 26.1, 27.0 and 27.1 High-Level Term “*Neisseria* infection.” Only cases associated with *Nm* were included.

Data were analyzed using summary tables of cumulative *Nm* cases generated from the Alexion pharmacovigilance database. Cumulative postmarketing exposure was calculated by adding the exposure in patient-months of new, continuing, and discontinued patients for all indications during the analysis period. Patient-months were converted to patient-years (PY) by dividing by 12; the cumulative PY exposure was calculated by adding the exposure from the given month to that of each previous month. Cumulative clinical trial exposure is based on exposure data from completed clinical trials and the enrollment/randomization schemes of ongoing trials. Rates of meningococcal infections and mortality were calculated as number of events per 100 PY. Other quantitative data were reported using descriptive statistics.

## Results

Exposure to eculizumab from March 16, 2007, through October 1, 2024, was 2457 PY in the clinical trial setting and 91,052 PY in the real-world setting. Cumulatively, 239 cases of meningococcal infections in patients treated with eculizumab were recorded, with seven (2.9%) cases from clinical trials and 232 (97.1%) cases from the real-world setting. Exposure to ravulizumab from December 21, 2018, through December 31, 2024, was 3287 PY in the clinical trial setting and 34,582 PY in the real-world setting. In patients treated with ravulizumab, there were 42 cases of meningococcal infections, with six (14.3%) cases from clinical trials and 36 (85.7%) cases from the real-world setting. The individual clinical trials with ≥1 meningococcal infection are listed in S1 Table.

**Fig 1:**
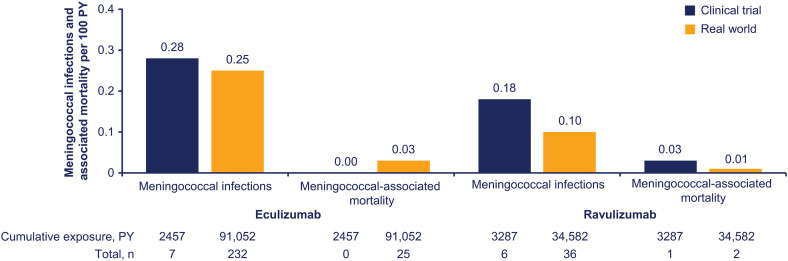
Cumulative rates of meningococcal infections and associated mortality per 100 PY among patients treated with eculizumab and ravulizumab in clinical trial and real-world settings. *PY* patient-years.

The cumulative rate of meningococcal infections in clinical trials was 0.28 and 0.18 per 100 PY in patients treated with eculizumab and ravulizumab, respectively. In the real-world setting, cumulative rates were 0.25 and 0.10 per 100 PY, respectively. The rates of meningococcal-associated mortality were ≤0.03 in both eculizumab- and ravulizumab-treated patients in clinical trials (0.00 and 0.03) and real-world settings (0.03 and 0.01) (Fig 1). Demographics and characteristics of patients with meningococcal infection are summarized in Table 1. Frequencies of meningococcal-related events are summarized in Table 2.

**Table 1:** Demographics, vaccination status, and identified serogroup among patients with meningococcal infection.

Patient Characteristic	Eculizumab- treated patients (n = 239)	Ravulizumab- treated patients (n = 42)
Age group, n (%)		
Pediatric (<18 years)	36 (15.1)	6 (14.3)
Adult (18–64 years)	167 (69.9)	28 (66.7)
Elderly (≥65 years)	9 (3.8)	2 (4.8)
Unknown	27 (11.3)	6 (14.3)
Sex, n (%)		
Female	119 (49.8)	21 (50.0)
Male	102 (42.7)	19 (45.2)
Not reported/unknown	18 (7.5)	2 (4.8)
Time to onset of meningococcal infection after first dose of eculizumab or ravulizumab, median (range), days	389 (1-3135)	223 (22-2346)
Vaccination status, n (%)		
All confirmed vaccinations	204 (85.4)	34 (81.0)
Unknown	35 (14.6)	8 (19.0)
Identified serogroup, n (%)		
A	3 (1.3)	0
B	46 (19.3)	4 (9.5)
C	9 (3.8)	0
E	2 (0.8)	2 (4.8)
W	9 (3.8)	2 (4.8)
X	1 (0.4)	0
Y	18 (7.5)	4 (9.5)
Y or W	2 (0.8)	1 (2.4)
Z	5 (2.1)	0
Non-groupable	16 (6.7)	4 (9.5)
Unknown/not reported/not tested	128 (53.6)	25 (59.5)^*^

*The culture result for one patient from Japan was reported as negative.

**Table 2:** Type and frequency of meningococcal-related events by *MedDRA* preferred term.

MedDRA preferred term, number of events	Eculizumab		Ravulizumab	
**Clinical trial**	**Real world**	**Clinical trial**	**Real world**
Encephalitis meningococcal	1	2	1	0
Meningitis meningococcal	2	53	0	10
Meningococcal bacteremia	0	34	0	1
Meningococcal infection	1	82	2	16
Meningococcal sepsis	3	95	3	11
Waterhouse-Friderichsen syndrome	0	4	0	0

Includes all events reported, there may be multiple preferred terms per case.

*MedDRA Medical Dictionary for Regulatory Activities*.

**Fig 2:**
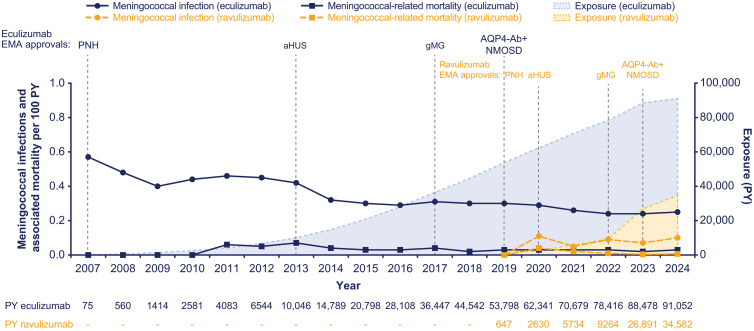
Rate of meningococcal infection and associated mortality per 100 PY among patients treated with eculizumab and ravulizumab in the real-world setting from 2007 to 2024. Grey vertical lines indicate EMA approval years. Data are inclusive of patients with PNH, aHUS, gMG, and AQP4-Ab+ NMOSD. The cutoff dates for these data were October 2024 (eculizumab) and December 2024 (ravulizumab). *aHUS* atypical hemolytic uremic syndrome, *AQP4-Ab+* anti-aquaporin-4 antibody-positive, *EMA* European Medicines Agency, *gMG* generalized myasthenia gravis, *NMOSD* neuromyelitis optica spectrum disorder, *PNH* paroxysmal nocturnal hemoglobinuria, *PY* patient-years.

In a real-world setting, cumulative meningococcal infection rates in patients treated with eculizumab decreased from ≈0.57 per 100 PY in 2007 to 0.25 per 100 PY in 2024. Associated mortality rates have been stable in the same time frame from ≈0.00 to 0.03 per 100 PY (Fig 2). In patients treated with ravulizumab, the cumulative meningococcal infection rates have remained stable from 0 per 100 PY in 2019 to 0.10 per 100 PY in 2024, and associated mortality rates remained stable in the same time frame from 0 to 0.01 per 100 PY, respectively (Fig 2). The number of meningococcal infections and associated deaths among eculizumab- and ravulizumab-treated patients in both clinical trials and real-world settings per indication are shown in S2 and S3 Tables.

## Discussion

In patients with complement deficiencies or patients who are treated with complement inhibitors, there is a risk of infection with the *Neisseria* species [15]. The global incidence of meningococcal infections is difficult to determine as the incidence rate varies geographically, temporally, and by age [17–20]. Although the true global burden of meningococcal infections is not known, the incidence of invasive meningococcal disease caused by *Nm* is approximately 433,000 to 1.2 million cases a year, including the most common clinical manifestations of meningitis and septicemia [17,19–21]. The incidence rate for meningococcal infections is estimated to be 1000- to 2000-fold higher in patients receiving a complement inhibitor compared with the general population [22]. Meningococcal infections are serious in nature and can be life-threatening or fatal if not recognized early and treated with appropriate antibiotics [23].

This report is the largest compilation of pharmacovigilance data on eculizumab and ravulizumab exposure to date, reflecting over 91,000 PY of eculizumab exposure and 34,500 PY of ravulizumab exposure in the real world. Long-term trends of meningococcal infections in patients receiving eculizumab or ravulizumab show that cumulative rates of meningococcal infections have decreased or remained stable over time with increasing exposure. There were a total of 25 and three meningococcal*-*associated deaths among patients treated with eculizumab and ravulizumab, respectively, reported across clinical trial and real-world experience. The case fatality rate of meningococcal infections in real-world settings for this analysis (≈5%−10%) is similar to the reported case fatality rate in the general population (≈6%−18%) [17,24,25]. The majority of events with fatal outcomes in this analysis were a consequence of delays in diagnosis and/or treatment of meningococcal infection, which is also likely the case in the general population [26].

Risk mitigation strategies are critical to reduce the morbidity and mortality related to meningococcal infections in patients when taking complement-inhibiting therapies. The findings from this analysis of pharmacovigilance surveillance data for eculizumab and ravulizumab suggest that the current mitigation measures, including meningococcal vaccination in accordance with local guidelines and use of antibiotic prophylaxis treatment when starting complement-inhibiting therapies, are generally effective, with only a small number of meningococcal infections reported in these data compared with the estimated number of meningococcal infections occurring each year. Although other complement inhibitors are approved for different indications, we are only able to speak to our experience with eculizumab and ravulizumab. Clinicians should rely on careful assessment of the potential benefits and risks of complement inhibitors for each patient and monitor patients to effectively mitigate the risk of meningococcal infection.

## Supporting information

S1 Table Eculizumab and ravulizumab clinical trials included in this analysis in which ≥1 meningococcal infection occurred. *NMOSD* neuromyelitis optica spectrum disorder.
(DOCX)

S2 Table Number of meningococcal infections and associated deaths among eculizumab-treated patients in clinical trial and real-world settings based on indication. *Includes indication no longer under study and off-label use in the real-world setting. ^†^Patient discontinued eculizumab 3 months before death; cause of death was fulminant brainstem relapse after having developed an infection and sepsis. *aHUS* atypical hemolytic uremic syndrome, *AQP4-Ab+* anti-aquaporin-4 antibody-positive, *gMG* generalized myasthenia gravis, *NMOSD* neuromyelitis optica spectrum disorder, *PNH* paroxysmal nocturnal hemoglobinuria.
(DOCX)

S3 Table Number of meningococcal infections and associated deaths among ravulizumab-treated patients in clinical trial and real-world settings based on indication. *One patient (Asian male; 62 years old; received ravulizumab throughout the study) died owing to meningococcal sepsis (strain unknown). He was enrolled in the ALXN1210-PNH-301 treatment-naive study ravulizumab-ravulizumab arm; no history of aplastic anemia; death occurred on study day 853. ^†^Two patients in the ALXN1210-NMO-307 (CHAMPION-NMOSD) trial experienced a meningococcal infection. Both cases were rapidly treated and resolved with no sequelae. ^‡^Includes indication no longer under study and unknown indication or off-label use in the real-world setting. *aHUS* atypical hemolytic uremic syndrome, *AQP4-Ab+* anti-aquaporin-4 antibody-positive, *gMG* generalized myasthenia gravis, *NMOSD* neuromyelitis optica spectrum disorder, *PNH* paroxysmal nocturnal hemoglobinuria.
(DOCX)

## References

[1] DeZern AE, Brodsky RA. Paroxysmal nocturnal hemoglobinuria: a complement-mediated hemolytic anemia. Hematol Oncol Clin North Am. 2015;29(3):479–94. doi: 10.1016/j.hoc.2015.01.005 26043387 PMC4695989

[2] Yoshida Y, Kato H, Nangaku M. Atypical hemolytic uremic syndrome. Ren Replace Ther. 2017;3(1). doi: 10.1186/s41100-016-0088-1

[3] Howard JF Jr. Myasthenia gravis: the role of complement at the neuromuscular junction. Ann N Y Acad Sci. 2018;1412(1):113–28. doi: 10.1111/nyas.13522 29266249

[4] Wingerchuk DM, Lucchinetti CF. Neuromyelitis optica spectrum disorder. N Engl J Med. 2022;387(7):631–9. doi: 10.1056/NEJMra1904655 36070711

[5] Rother RP, Rollins SA, Mojcik CF, Brodsky RA, Bell L. Discovery and development of the complement inhibitor eculizumab for the treatment of paroxysmal nocturnal hemoglobinuria. Nat Biotechnol. 2007;25(11):1256–64. doi: 10.1038/nbt1344 17989688

[6] Sheridan D, Yu Z-X, Zhang Y, Patel R, Sun F, Lasaro MA, et al. Design and preclinical characterization of ALXN1210: A novel anti-C5 antibody with extended duration of action. PLoS One. 2018;13(4):e0195909. doi: 10.1371/journal.pone.0195909 29649283 PMC5897016

[7] Pouw RB, Ricklin D. Tipping the balance: intricate roles of the complement system in disease and therapy. Semin Immunopathol. 2021;43(6):757–71. doi: 10.1007/s00281-021-00892-7 34698894 PMC8547127

[8] Bouwman HB, Guchelaar H-J. The efficacy and safety of eculizumab in patients and the role of C5 polymorphisms. Drug Discov Today. 2024;29(9):104134. doi: 10.1016/j.drudis.2024.104134 39111540

[9] Meisel A, Annane D, Vu T, Mantegazza R, Katsuno M, Aguzzi R, et al. Long-term efficacy and safety of ravulizumab in adults with anti-acetylcholine receptor antibody-positive generalized myasthenia gravis: results from the phase 3 CHAMPION MG open-label extension. J Neurol. 2023;270(8):3862–75. doi: 10.1007/s00415-023-11699-x 37103755 PMC10134722

[10] Kulasekararaj AG, Griffin M, Langemeijer S, Usuki K, Kulagin A, Ogawa M, et al. Long-term safety and efficacy of ravulizumab in patients with paroxysmal nocturnal hemoglobinuria: 2-year results from two pivotal phase 3 studies. Eur J Haematol. 2022;109(3):205–14. doi: 10.1111/ejh.13783 35502600 PMC9546219

[11] Barbour T, Scully M, Ariceta G, Cataland S, Garlo K, Heyne N, et al. Long-term efficacy and safety of the long-acting complement C5 inhibitor ravulizumab for the treatment of atypical hemolytic uremic syndrome in adults. Kidney Int Rep. 2021;6(6):1603–13. doi: 10.1016/j.ekir.2021.03.884 34169200 PMC8207473

[12] Pittock SJ, Barnett M, Bennett JL, Berthele A, de Sèze J, Levy M, et al. Ravulizumab in aquaporin-4-positive neuromyelitis optica spectrum disorder. Ann Neurol. 2023;93(6):1053–68. doi: 10.1002/ana.26626 36866852

[13] Schneider MC, Exley RM, Ram S, Sim RB, Tang CM. Interactions between Neisseria meningitidis and the complement system. Trends Microbiol. 2007;15(5):233–40. doi: 10.1016/j.tim.2007.03.005 17398100

[14] Jayaraman A, Walachowski S, Bosmann M. The complement system: a key player in the host response to infections. Eur J Immunol. 2024;54(11):e2350814. doi: 10.1002/eji.202350814 39188171 PMC11623386

[15] Lewis LA, Ram S. Meningococcal disease and the complement system. Virulence. 2014;5(1):98–126. doi: 10.4161/viru.26515 24104403 PMC3916388

[16] Koelman DLH, Brouwer MC, van de Beek D. Targeting the complement system in bacterial meningitis. Brain. 2019;142(11):3325–37. doi: 10.1093/brain/awz222 31373605 PMC6821383

[17] Guedes S, Bricout H, Langevin E, Tong S, Bertrand-Gerentes I. Epidemiology of invasive meningococcal disease and sequelae in the United Kingdom during the period 2008 to 2017 - a secondary database analysis. BMC Public Health. 2022;22(1):521. doi: 10.1186/s12889-022-12933-3 35296287 PMC8928586

[18] Pardo de Santayana C, Tin Tin Htar M, Findlow J, Balmer P. Epidemiology of invasive meningococcal disease worldwide from 2010-2019: a literature review. Epidemiol Infect. 2023;151:e57. doi: 10.1017/S0950268823000328 37052295 PMC10126893

[19] Shen S, Findlow J, Peyrani P. Global epidemiology of meningococcal disease-causing serogroups before and after the COVID-19 pandemic: a narrative review. Infect Dis Ther. 2024;13(12):2489–507. doi: 10.1007/s40121-024-01063-5 39509011 PMC11582116

[20] Bosis S, Mayer A, Esposito S. Meningococcal disease in childhood: epidemiology, clinical features and prevention. J Prev Med Hyg. 2015;56(3):E121-4. 26788732 PMC4755120

[21] GBD 2019 Meningitis Antimicrobial Resistance Collaborators. Global, regional, and national burden of meningitis and its aetiologies, 1990-2019: a systematic analysis for the Global Burden of Disease Study 2019. Lancet Neurol. 2023;22(8):685–711. doi: 10.1016/S1474-4422(23)00195-3 37479374 PMC10356620

[22] McNamara LA, Topaz N, Wang X, Hariri S, Fox L, MacNeil JR. High risk for invasive meningococcal disease among patients receiving eculizumab (Soliris) despite receipt of meningococcal vaccine. MMWR Morb Mortal Wkly Rep. 2017;66(27):734–7. doi: 10.15585/mmwr.mm6627e1 28704351 PMC5687588

[23] Nadel S. Treatment of meningococcal disease. J Adolesc Health. 2016;59(2 Suppl):S21–8. doi: 10.1016/j.jadohealth.2016.04.013 27449146

[24] Parikh SR, Campbell H, Bettinger JA, Harrison LH, Marshall HS, Martinon-Torres F, et al. The everchanging epidemiology of meningococcal disease worldwide and the potential for prevention through vaccination. J Infect. 2020;81(4):483–98. doi: 10.1016/j.jinf.2020.05.079 32504737

[25] CDC. Increase in invasive serogroup Y meningococcal disease in the United States. [Accessed 2025 January 7]. https://www.cdc.gov/han/2024/han00505.html#print

[26] Ciftci E, Ocal D, Somer A, Tezer H, Yilmaz D, Bozkurt S, et al. Current methods in the diagnosis of invasive meningococcal disease. Front Pediatr. 2025;13:1511086. doi: 10.3389/fped.2025.1511086 40330073 PMC12053261

