# Supplementary material for: Eculizumab and ravulizumab clinical trial and real-world pharmacovigilance of meningococcal infections across indications
Source: PLoS One. 2025 Sep 12;20(9):e0332073. doi: 10.1371/journal.pone.0332073 (PMC12431217; doi:10.1371/journal.pone.0332073)
Supplement: S1 Table — NMOSD neuromyelitis optica spectrum disorder. (DOCX) [file pone.0332073.s001.docx]

## S1 Table. Eculizumab and ravulizumab clinical trials included in this analysis in which ≥1 meningococcal infection occurred.

| **Treatment** | **Clinical trial number** | **Alexion study ID** | **Title** | **Phase** |
| --- | --- | --- | --- | --- |
| Eculizumab | NCT01194973 | C10-004 | An Open-label, Multi-center Clinical Trial of Eculizumab in Adult Patients With Atypical Hemolytic-uremic Syndrome | 2 |
|  | NCT02301624 | ECU-MG-302 | Extension Study of ECU-MG-301 to Evaluate Safety and Efficacy of Eculizumab in Refractory Generalized Myasthenia Gravis | 3 |
|  | NCT04155424 | ECU-NMO-303 | A Study of the Safety and Activity of Eculizumab in Pediatric Participants With Relapsing Neuromyelitis Optica Spectrum Disorder | 2/3 |
|  | NCT00122317 | E05-001 | Extension Study of Eculizumab in Patients With Transfusion Dependent Paroxysmal Nocturnal Hemoglobinuria (PNH) | 3 |
| Ravulizumab | NCT02946463 | ALXN1210-PNH-301 | ALXN1210 (Ravulizumab) Versus Eculizumab in Complement Inhibitor Treatment-Naïve Adult Participants With Paroxysmal Nocturnal Hemoglobinuria (PNH) | 3 |
|  | NCT02598583 | ALXN1210-PNH-103 | Dose-Escalation Study of ALXN1210 IV in Participants With Paroxysmal Nocturnal Hemoglobinuria (PNH) | 1/2 |
|  | NCT02605993 | ALXN1210-PNH-201 | Open-label, Multiple Ascending Dose Study of Ravulizumab (ALXN1210) in Participants With Paroxysmal Nocturnal Hemoglobinuria (PNH) | 2 |
|  | NCT04201262 | ALXN1210-NMO-307 | An Efficacy and Safety Study of Ravulizumab in Adult Participants with NMOSD | 3 |

*NMOSD* neuromyelitis optica spectrum disorder.
