# Supplementary material for: Eculizumab and ravulizumab clinical trial and real-world pharmacovigilance of meningococcal infections across indications
Source: PLoS One. 2025 Sep 12;20(9):e0332073. doi: 10.1371/journal.pone.0332073 (PMC12431217; doi:10.1371/journal.pone.0332073)
Supplement: S2 Table — *Includes indication no longer under study and off-label use in the real-world setting. †Patient discontinued eculizumab 3 months before death; cause of death was fulminant brainstem relapse after having developed an infection and sepsis. aHUS atypical hemolytic uremic syndrome, AQP4-Ab+ anti-aquaporin-4 antibody-positive, gMG generalized myasthenia gravis, NMOSD neuromyelitis optica spectrum disorder, PNH paroxysmal nocturnal hemoglobinuria. (DOCX) [file pone.0332073.s002.docx]

## S2 Table. Number of meningococcal infections and associated deaths among eculizumab-treated patients in clinical trial and real-world settings based on indication.

| **Indication** | **Clinical trial** | | **Real world** | |
| --- | --- | --- | --- | --- |
|  | Meningococcal infection, n | Meningococcal-associated deaths, n | Meningococcal infection,  n | Meningococcal-associated deaths, n |
| PNH | 2 | 0 | 115 | 19 |
| aHUS | 2 | 0 | 72 | 3 |
| gMG | 1 | 0 | 10 | 1 |
| AQP4-Ab+ NMOSD | 1 | 0 | 5 | 1^†^ |
| Other* | 1 | 0 | 19 | 1 |
| Unknown | 0 | 0 | 11 | 0 |
| Total | 7 | 0 | 232 | 25 |

*Includes indication no longer under study and off-label use in the real-world setting. ^†^Patient discontinued eculizumab 3 months before death; cause of death was fulminant brainstem relapse after having developed an infection and sepsis.
*aHUS* atypical hemolytic uremic syndrome, *AQP4-Ab+* anti-aquaporin-4 antibody-positive, *gMG* generalized myasthenia gravis, *NMOSD* neuromyelitis optica spectrum disorder, *PNH* paroxysmal nocturnal hemoglobinuria.
