# Supplementary material for: Eculizumab and ravulizumab clinical trial and real-world pharmacovigilance of meningococcal infections across indications
Source: PLoS One. 2025 Sep 12;20(9):e0332073. doi: 10.1371/journal.pone.0332073 (PMC12431217; doi:10.1371/journal.pone.0332073)
Supplement: S3 Table — *One patient (Asian male; 62 years old; received ravulizumab throughout the study) died owing to meningococcal sepsis (strain unknown). He was enrolled in the ALXN1210-PNH-301 treatment-naive study ravulizumab-ravulizumab arm; no history of aplastic anemia; death occurred on study day 853. †Two patients in the ALXN1210-NMO-307 (CHAMPION-NMOSD) trial experienced a meningococcal infection. Both cases were rapidly treated and resolved with no sequelae. ‡Includes indication no longer under study and unknown indication or off-label use in the real-world setting. aHUS atypical hemolytic uremic syndrome, AQP4-Ab+ anti-aquaporin-4 antibody-positive, gMG generalized myasthenia gravis, NMOSD neuromyelitis optica spectrum disorder, PNH paroxysmal nocturnal hemoglobinuria. (DOCX) [file pone.0332073.s003.docx]

## S3 Table. Number of meningococcal infections and associated deaths among ravulizumab-treated patients in clinical trial and real-world settings based on indication.

| **Indication** | **Clinical trial** | | **Real world** | |
| --- | --- | --- | --- | --- |
|  | Meningococcal infection,  n | Meningococcal-associated deaths, n | Meningococcal infection,  n | Meningococcal-associated deaths, n |
| PNH | 4 | 1* | 22 | 2 |
| aHUS | 0 | 0 | 8 | 0 |
| gMG | 0 | 0 | 3 | 0 |
| AQP4-Ab+ NMOSD | 2^†^ | 0 | 0 | 0 |
| Other^‡^ | - | - | 3 | 0 |
| Total | 6 | 1 | 36 | 2 |

*One patient (Asian male; 62 years old; received ravulizumab throughout the study) died owing to meningococcal sepsis (strain unknown). He was enrolled in the ALXN1210-PNH-301 treatment-naive study ravulizumab-ravulizumab arm; no history of aplastic anemia; death occurred on study day 853. ^†^Two patients in the ALXN1210-NMO-307 (CHAMPION-NMOSD) trial experienced a meningococcal infection. Both cases were rapidly treated and resolved with no sequelae. ^‡^Includes indication no longer under study and unknown indication or off-label use in the real-world setting.
*aHUS* atypical hemolytic uremic syndrome, *AQP4-Ab+* anti-aquaporin-4 antibody-positive, *gMG* generalized myasthenia gravis, *NMOSD* neuromyelitis optica spectrum disorder, *PNH* paroxysmal nocturnal hemoglobinuria.
